# Monocyte Secretion of *β*-Hexosaminidase in Patients With Obstructive Jaundice

**DOI:** 10.1155/1993/40894

**Published:** 1993

**Authors:** W. G. Jiang, M. C. A. Puntis

**Affiliations:** Department of Surgery University of Wales College of Medicine Cardiff CF4 4XN United Kingdom

## Abstract

Monocyte hydrolases are harmful when secreted inappropriately. In this study we have investigated the levels of one of the hydrolases, *β*-hexosaminidase in patients with obstructive jaundice. These patients showed markedly elevated plasma levels, and their monocytes show increased spontaneous secretion and total enzyme content. The plasma enzyme levels correlate with monocyte enzyme content as well as bile salt, and bilirubin levels, the high levels may also reflect Kupffer cell damage, as these cells clear the enzyme. Compared with controls monocytes from jaundiced patients show reduced enzyme secretion after PMA stimulation, *in vitro*, and unchanged secretion after zymozan stimulation. There is a difference between plasma enzyme levels in benign and malignant patients but this does not provide a clear distinction between the two groups. We conclude that patients with obstructive jaundice have increased blood level of *β*-hexosaminidase, and that activated monocytes partly contribute to this change.


*HPB Surgery*, 1993, Vol. 7, pp. 15–23
Reprints available directly from the publisher
Photocopying permitted by license only

© 1993 Harwood Academic Publishers GmbH
Printed in the United States of America

### MONOCYTE SECRETION OF β-HEXOSAMINIDASE IN PATIENTS WITH OBSTRUCTIVE JAUNDICE

W.G.JIANG and M.C.A.PUNTIS*
*Department of Surgery, University of Wales College of Medicine, Cardiff CF4 4XN, UK*

*(Received 19 October 1992)*

Monocyte hydrolases are harmful when secreted inappropriately. In this study we have investigated the levels of one of the hydrolases. β-hexosaminidase in patients with obstructive jaundice. These patients showed markedly elevated plasma levels, and their monocytes show increased spontaneous secretion and total enzyme content. The plasma enzyme levels correlate with monocyte enzyme content as well as bile salt, and bilirubin levels, the high levels may also reflect Kupffer cell damage, as these cells clear the enzyme. Compared with controls monocytes from jaundiced patients show reduced enzyme secretion after PMA stimulation, *in vitro*, and unchanged secretion after zymozan stimulation. There is a difference between plasma enzyme levels in benign and malignant patients but this does not provide a clear distinction between the two groups. We conclude that patients with obstructive jaundice have increased blood level of β-hexosaminidase, and that activated monocytes partly contribute to this change.

KEY WORDS: β-hexosaminidase, monocytes, obstructive jaundice, bile salts

### INTRODUCTION

β-hexosaminidase (EC 3.2.1 52) is one of the lysosomal hydrolases that is critically deficient in Tay-Sachs and Sandoff syndrome[1,2]. This enzyme has been found to be increased in several other diseases: e.g. diabetes mellitus[3], sepsis[4], enterocolitis[5], and some malignancies[6,7,8,9]. Hultberg[10] reported in 1981 that this enzyme was also increased in the serum from obstructive jaundice patients and together with other researchers[11] suggested this might be due to the decreased clearance by Kupffer cells in these disease conditions.

Monocytes are an important component of the reticuloendothelial system. This cell population is known to secrete many substances including proteins, enzymes, cytokines, lipid metabolites and other immune regulators. It has been shown that monocytes/macrophages secrete lysosomal enzymes spontaneously[12] and in response to stimuli[13,14]. Previous work has shown that patients with obstructive jaundice have altered immune responses[15,16,17,18]. The monocyte function however in jaundice has not yet been fully studied. The relationship between monocyte secretion of lysosomal enzymes and plasma β-hexosaminidase level in obstructive jaundice is poorly understood.

In this study we have investigated the monocyte β-hexosaminidase secretion under stimulated and non-stimulated conditions and its relation with plasma β-hexosaminidase and bile salts levels.

### MATERIALS AND METHODS

### *Patients and Controls*

Fifty eight patients with obstructive jaundice were included in the study. Twenty nine had a cholangiocarcinoma, 7 had pancreatic carcinoma and 1 gallbladder cancer, the remaining 21 had benign disease which was due to gallstones in 19 and a benign biliary stricture in two. Forty-two controls were either non-jaundiced patients admitted for elective surgery ($n=32$) or normal volunteers ($n=10$). The patients in the control group comprised 20 with benign diseases (14 with gallstones, 5 with inguinal hernias and 1 with haemorrhoids) and 13 with malignant disease (colon cancer with or without liver metastases). Patients with sepsis were excluded. The median age for the jaundiced patients was 64.7y (IQR 57.3-74.8) and for the controls 53.8y (IQR 42.5-70.5). Informed consent to participate in the study was obtained from all the patients. Blood was taken immediately after admission, before any procedures were carried out.

### *Cell Preparation*

All materials were purchased from Sigma unless otherwise stated. 20mls peripheral blood were taken into a Sterilin bottle (Sterilin, England) containing 200U heparin. Blood was put immediately onto ice and processed, at 4°C, as soon as possible (within 60mins). Plasma was obtained after centrifugation of blood at 1500 rpm for 20 minutes it was then stored at -80°C until needed. Mononuclear cells (MNC) were obtained by Ficoll-Hypaque separation. A small portion of the resulting cell suspension was used for non-specific esterase staining to estimate the monocyte proportion in the MNC preparation, this was: 29% (IQR 25%-36%) in jaundice and 27% (25%-31%) in controls. MNC's at $2 \times 10^6$ were then added to a multiwell plate (NUNC, Denmark) at 100$\mu$l/well and allowed to adhere to the plastic surface at 37°C in 5% $CO_2$ for 60 minutes. Non adherent cells were then washed off and the monocytes were re-suspended in RPMI 1640 with 2.0% bovine serum albumin (BSA, fraction V, Boehringer Mannheim GmbH, Germany). Serum was avoided in cell culture because it contains a high concentration of $\beta$-hexosaminidase.

### *Cell Stimulation*

Aliquots of cells in triplicate were then treated with (i) culture medium only for estimation of spontaneous secretion, with (ii) phorbol myristate acetate (PMA) at a concentration 5.0 $\mu$g/ml, or with (iii) opsonised zymozan particles at 0.5 mg/ml made up as follows; 10mg zymozan was heat inactivated at 100°C for 10 minutes and washed, 2.0 ml pooled human serum was added to the particles and incubated at 37°C for 30 minutes after washing 10 times at 2000 rpm for 10 minutes the particles were re-suspended in culture medium and stored at -80°C. Some cells were also treated by 10% Triton X100 to cause the release of the total enzyme content of the monocytes. After 4 hours culture with either medium, one of the stimulants or with Triton the plates were centrifuged at 1000 rpm for 20 minutes. Supernatants were harvested with a multichannel pipette and stored at -80°C before analysis. Cell viability was checked by the trypan blue exclusion test before and after experiments and both were $>93\%$.

*Enzyme Assay*

β-hexosaminidase was assayed using a substrate Nitrophenol-β-N-acetyl-glucosaminide[6,19] dissolved in citrate buffer (pH 4.5) at 3.75 mM. The substrate was added to samples and incubated at 37°C for 4 hours in the case of monocyte supernatant or, because of the higher concentration of enzyme for only 30 minutes in the case of Plasma. In both systems the reaction was terminated by adding glycine buffer (pH 10.5). The coloured product was measured on a Titertek Multiscan (Flow, UK) with a standard filter (wavelength 405nm) and was compared with the internal nitrophenol standard. 1U enzyme per litre is the activity to produce 1μM nitrophenol per min. Monocyte spontaneous and stimulated enzyme secretion were expressed as a percentage of the total enzyme content of the cell that is released by Triton treatment. Monocyte total enzyme content is shown as Units per million monocytes.

*Other Assays*

Plasma bile salts were measured with a commercial kit Enzabile from Nycomed AS., Oslo, Norway. The assay was carried out according to the manufacturers instructions and the concentrations were calculated from the standard bile salts provided with the kit. Plasma bilirubin, aspartate aminotransferase, alkaline phosphatase and albumin were measured at the same time by routine laboratory methods. The results are expressed as median (inter quartile range, IQR) and statistical tests used were Mann-Whitney U test and Spearman rank correlation test.

### RESULTS

### Monocyte β-hexosaminidase Secretion

*Spontaneous secretion*
Monocytes from obstructive jaundice patients have an increased spontaneous secretion of β-hexosaminidase at 17.4 (14.3-21.3)% compared with only 8.1 (3.5-14.7)% in controls, $p<0.01$ (secretion is expressed as a percentage of the cells' total enzyme contents). This increase was especially marked in benign jaundiced patients as shown in Table 1.

*PMA stimulated secretion*
In contrast to the spontaneous secretion, monocytes from jaundiced patients greatly reduced their enzyme secretion after PMA stimulation to 7.9 (2.6-16.5)% compared with 15.5 (8.3-22.0)% in controls, ($p<0.01$). Analysis of the subgroups of patients revealed that this change was even more pronounced in the malignant patients. This data is shown in Table 2.

*Zymozan stimulated secretion*
Stimulation with opsonised zymozan particles caused control monocytes to secrete more lysosomal enzyme and this is consistent with the work of others [13,14] confirming that opsonised zymozan particles are the most potent stimulator of

**Table 1** Enzyme secretion is expressed as a percentage of the total quantity of enzyme released from an equal number of monocytes by treatment with Triton X100. Increased secretion is especially high in benign jaundiced patients

| *Spontaneous enzyme secretion from control and jaundiced patients* | | | |
|---|---|---|---|
| | *Benign* | *Malignant* | *p value* |
| Control | 6.4% (2.7-12.8)% | 11.4% (7.2-19.6)% | =0.15 |
| Jaundice | 32.5% (20.6-62.7)% | 16.1% (12.7-19.8)% | <0.01 |
| p value | <0.01 | =0.01 | |

**Table 2** Enzyme seretion is expressed as a percentage of the total quantity of enzyme released from an equal number of monocytes by treatment with Triton X100. The secretion from malignant jaundiced patients is significantly reduced compared with both controls and benign jaundiced patients

| *PMS stimulated lysosomal enzyme secretion from monocytes* | | | |
|---|---|---|---|
| | *Benign* | *Malignant* | *p value* |
| Control | 15.7% (5.8-23.5)% | 15.3% (11.8-20.0)% | 0.77 |
| Jaundice | 10.2% (4.5-17.1)% | 7.4% (0.8-12.6)% | <0.05 |
| p value | =0.17 | <0.01 | |

monocyte lysosomal enzyme secretion. Zymozan stimulation of monocytes from jaundiced patients showed a response similar to that in controls with 27.8 (16.8-39.0)% in jaundice compared with 29.5 (17.3-42.8)% in controls, ($p = 0.77$). The responses were the same in benign and malignant disease, the data is shown in Table 3.

*Monocyte total enzyme contents*

As measured in the supernatant from Triton lysed monocytes the total enzyme content from jaundiced patients was 0.46 (0.32-0.56) U/million cells which was higher than controls 0.28 (0.18-0.36) U/million cells with $p < 0.01$. Analysis of subgroups of patients showed a significant difference between the benign and malignant jaundiced patients, but not between other groups, this data is shown in Table 4.

### Plasma $\beta$-Hexosaminidase and Bile Salt Levels

*$\beta$-Hexosaminidase*

The plasma enzyme levels were significantly increased in obstructive jaundice patients, 39.7 (28.4-54.5) U/l compared with controls, 13.4 (9.5-19.7) U/l.

**Table 3** Enzyme secretion is expressed as a percentage of the total quantity of enzyme released from an equal number of monocytes by treatment with Triton X100. All groups respond in the same way to zymozan

| *Zymosan stimulated lysosomal enzyme secretion from monocytes* | | | |
|---|---|---|---|
| | *Benign* | *Malignant* | *p value* |
| Control | 28.0% (14.1-36.8)% | 34.6% (17.3-42.8)% | =0.21 |
| Jaundice | 29.8% (15.2-47.9)% | 27.7% (17.0-34.9)% | =0.42 |
| p value | =0.67 | =0.07 | |

**Table 4** β-Hexosaminidase is expressed as Units per million cells. The monocytes from malignant jaundice patients release most enzyme in response to Triton X100

| *Total lysosomal enzyme release from monocytes* | | | |
|---|---|---|---|
| | *Benign* | *Malignant* | *p value* |
| Control | 0.26 (0.14-0.34) | 0.34 (0.20-0.58) | =0.08 |
| Jaundice | 0.34 (0.2-0.48) | 0.52 (0.34-0.66) | <0.01 |
| p value | =0.0.25 | =0.14 | |

($p<0.001$). The differences between benign controls (12.2(9.5-16.7) U/l) and benign jaundiced (31.8(28.2-47.6) U/l), and also between malignant controls (18.9(3.7-25.5) U/l) and malignant jaundiced (46.7(30.4-63.5) U/l) all reached statistical significance. There was also a difference between the benign and malignant jaundiced patients, but no difference between control groups was seen.

*Bile salts*

As expected plasma bile salt levels in jaundiced patients, 61.5 (14.5-101.5) μM/l were greatly increased compared with controls, 8.9 (4.8-16.5) μM/l, $p<0.001$. Statistically significant differences were also seen in analysing the benign subgroup 6.2(2.0-11.9) μM/l in control and 50.8(14.2-68.0) μM/l in jaundice and the malignant subgroup 15.8(12.3-37.6) μM/l in control and 81.3(15.3-108.2) μM/l in jaundice.

*Plasma biochemistry*

Plasma biochemistry from patients and controls is summarised in Table 5. The controls all had values within the normal range for this institution.

*The relationships between the monocyte lysosomal secretions, plasma enzyme levels, bile salts and biochemistry in jaundice patients*

As shown in Table 6, the increased monocyte total enzyme contents and decreased

**Table 5**

*Plasma Biochemistry*

| | *Control* | *Jaundice* |
|---|---|---|
| Bilirubin mM/l | 9.0 (6.7-14.0) | 136.0 (51.0-265.2) |
| Alk.phos. IU/l | 94.5 (85.2-112.0) | 592.0 (370.3-848.8) |
| AST IU/l | 20.0 (16.0-28.5) | 74.0 (51.0-142.0) |
| Albumin g/l | 42.5 (40.7-44.3) | 35.5 (32.0-38.5) |

Data expressed as median (IQR)

**Table 6**

*The correlation coefficients between monocyte hexosaminidase activity and liver function*

| | *Spont.* | *PMA* | *Zymozan* | *LPS* | *Total* | *Plasma* |
|---|---|---|---|---|---|---|
| Bile salts | 0.07 | −0.28 | −0.21 | 0.23 | 0.28 | 0.44* |
| Bilirubin | −0.06 | −0.47* | −0.18 | −0.06 | 0.39* | 0.35* |
| AST | −0.15 | −0.13 | −0.24 | 0.08 | 0.06 | −0.01 |
| Alk. Phos. | −0.06 | −0.10 | −0.24 | 0.05 | 0.19 | 0.35* |
| Albumin | −0.16 | 0.19 | −0.23 | −0.14 | −0.18 | −0.03 |

* = Significant correlation $p$ <0.05.

response to PMA stimulation have a close relationship with the degree of jaundice (correlation indes $r = 0.39$, $p = 0.01$ and $r = 0.47$, $p < 0.01$ respectively).

Plasma $\beta$-hexosaminidase level was also positively correlated with monocyte total enzyme contents ($r = 0.31$), bile sale levels ($r = 0.44$), alkaline phosphatase ($r = 0.35$) and bilirubin ($r = 0.35$)

### DISCUSSION

$\beta$-hexosaminidase is one of the monocyte lysosomal hydrolases. This enzyme is cleared from the circulation by Kupffer cells in the liver[20,21], by the kidney[22] and some other tissues. In blood however, this enzyme exists as its precursor form and the mode of clearance of the precursor is not clear[23]. Hultberg[5] in 1981 reported that the concentration of this enzyme increased in the blood of patients with obstructive jaundice. This enzyme has also been found to be increased in animals after hepatectomy[24], or with sepsis[4]. These studies suggested that the increases were due to damaged Kupffer cell clearance rather than increased production. The correlations between plasma $\beta$-hexosaminidase level and bile salt levels has also been previously reported in patients with intrahepatic cholestasis[11]. We confirm from our study that the blood levels of $\beta$-hexosaminidase are elevated in both benign and malignant jaundiced patients. Our data also shows that plasma β-hexosaminidase is positively correlated with bile salt and bilirubin levels. The plasma β-hexosaminidase level has been reported to be a marker to distinguish between patients with benign or malignant jaundice[25]. Our data only partly supports this conclusion as there is a considerable overlap between the two groups.

Jaundiced monocytes have an increased total enzyme content (Table 4) and this is correlated with plasma β-hexosaminidase level suggesting that monocytes contribute in part to the increased plasma enzyme level in addition to the depressed Kupffer cell clearance of the enzyme. Therefore the increased plasma β-hexosaminidase is not only a measure for the function of the fixed cells of the reticuloendothelial system[26,27] but also partly reflects peripheral monocyte activation. The change in monocyte total enzyme content in jaundiced patients is different from that reported by Holdstock *et al.*[28], where the enzyme levels were found to be reduced in cirrhotic patients. The changes seen here are similar to those in the small number of jaundiced patients in Holdstock's report.

Monocytes secrete some lysosomal enzyme spontaneously (Table 1) and increased amounts in response to stimulation. Indeed we have shown that PMA (Table 2) and opsonised zymozan particles (Table 3) can stimulate β-hexosaminidase secretion from control monocytes however PMA has an inhibitory effect on release of enzyme from jaundiced cells (that is, a lower proportion of the totaly enzyme content of the cell is released; although the content is higher in jaundice). In the case of zymozan stimulation jaundice and control cells release the same proportions of their respective totals. It is known that zymozan has its effects on monocyte lysosomal secretion via mannose-like receptors on the cells. Our data may suggest that the capacity for phagocytosis of these particles remains intact in obstructive jaundice. Indeed previous work in this laboratory has shown that monocytes from jaundiced patients phagocytose particles (bacteria and latex beads) normally (Puntis, M.C.A. data presented at the 2nd World Congress of HPB Surgery 1988). These results are different from some other reported investigations[29] which may be due to the material used in the study. Phorbol myristate acetate is a kinase C activator and its effects involve a cell surface receptor and intracellular mechanisms[30]. The reason for the inhibitory effect on the release of β-hexosaminidase from jaundiced monocytes in response to this stimulus is not clear at present. Monocytes from jaundiced patients also show increased spontaneous secretion of β-hexosaminidase. Two possible explanations for this are: (i) membrane fatty acids may be modified, as it has been shown that in jaundiced patients essential fatty acids are deficient[31]. The membrane fatty acids are an important component of several cell functions involving the cell surface and intracellular mechanisms[32]. We have previously reported that *in vitro* supplementation with n-6 fatty acids may inhibit the spontaneous secretion of this enzyme in obstructive jaundice[33]. Essential fatty acids and their metabolites are involved in the immune response[34] and therefore may play an important role in the altered monocyte function that we report here in jaundiced patients. (ii) Monocyte secretion of cytokines is modified in jaundiced patients. We have already reported that tumour necrosis factor (TNF) and interleukin-1 can stimulate monocyte lysosomal enzyme secretion[35] and TNF release by monocytes is increased in jaundiced patients[36].

The effects *in vivo* of the altered lysosomal enzyme release in jaundice are not yet clear. It is known that monocyte lysosomal enzyme may cause tissue damage in amoebiasis of the gut[37] and inflammatory bowel disease[38], also $\beta$-hexosaminidase from other sources such as mucosa can cause lesions in other tissues[39]. We suggest that the increased spontaneous secretion in jaundice may also be harmful to patients. In the presence of infection, for example, monocyte/macrophages may fail to respond adequately and this again may well be harmful to patients.

*Acknowledgements*

We thank Scotia Pharmaceuticals Ltd. for their support of this work. We also thank Dr M.B.Hallett for constructive discussions.

### *REFERENCES*

1. Okada, S. and O'Brien, J.S. (1969) Tay-sachs disease: generalised absence of a beta-D-N-acetylhexosaminidase component. *Science*, **165**, 698–700
2. Sandoff, D. (1969) Variation of $\beta$-N-acetylhexosaminidase-pattern in Tay-sachs disease. *FEBS Lett*, **4**, 351–354
3. Whiting, P.H., Ross, I.S. and Borthwick, L.J. (1979) N-acetyl-beta-D-hexosaminidase levels and diabetic microangiography. *Clin.Chim.Acta*, **97**, 191–195
4. Tonnesen, T., Andersen, P. and Guttler, F. (1988) Highly increased levels of serum $\beta$-hexosaminidase, arylsulphatase A and $\beta$-galactosidase in a patient with sepsis. *J.Inher.Metab.Dis.*, **11**, 428–429
5. Shattuck, K.E., Richardson, C.J., Rassin, D.K. and Lobe, T.E. (1987) Evaluation of hexosaminidase activity as a potential biochemical marker in serum for necrotizing enterocolitis. *J.Pediatr.Gastroenterol.Nutr*, **6**, 234–237
6. Tsao, D., Freeman, H.J. and Kim, Y.S. (1979) $\beta$-hexosaminidase isoenzymes in tissues, cultured cells and media from human fetal intestine and colonic adenocarcinoma. *Cancer Res.*, **39**, 3405–3410
7. Alhadeff, J.A. and Holzinger, R.T. (1982) Presence of an atypical thermolabile species of $\beta$-hexosaminidase B in metastatic-tumour tissue of human liver. *Biochem.J.*, **201**, 95–99
8. Brattain, M.G., Kimball, P.M., Durant, J.R., Pretlow, II, T.G., Smith, D., Carpenter, J. and Marks, M. (1979) Urinary hexosaminidase in patients with lung carcinoma. *Cancer*, **44**, 2267–2272
9. Weber, A., Poenaru, L., Lafarge, C. and Schapira, F. (1973) Modification of hexosaminidase isozymes in rat hepatoma. *Cancer Res.*, **33**, 1925–1930
10. Hultberg, B., Isaksson, A. and Jansson, L. (1981) $\beta$-hexosaminidase in serum from patients with cirrhosis and cholestasis. *Enzyme*, **26**, 296–300
11. Scapa, E., Novis, B.H., Loewenstein, M., Thomas, P. and Zamcheck, N. (1988) Serum $\beta$-N-acetyl hexosaminidase and bile acid levels in patients with benign and malignant biliary obstruction. *Dig.Dis.Sci.*, **33**, 189–192
12. Jessup, W. and Dean, R.T. (1980) Spontaneous lysosomal enzyme secretion by a murine macrophage-like cell line. *Biochem.J.*, **190**, 847–850
13. Bourbouze, R., Raddi, F., Dameron, G., Hali-Miraftab, H., Loko, F. and Vilde, J.L. (1991) N-acetyl-$\beta$-D-glucosaminidase (NAG) isoenzymes release from human monocyte-derived macrophages in response to zymozan and human recombinant interferon-$\alpha$. *Clin.Chim.Acta.*, **199**, 185–194
14. Leoni, P. and Dean, R.T. (1983) Mechanisms of lysosomal enzyme secretion by human monocytes. *Biochim.Biophys.Acta*, **762**, 378–389
15. Halpeax, B.N., Biozzi, G., Nicol, T. Bilbby, D.L.J. (1957) Effect of experimental biliary obstruction on the phagocytic activity of the reticulo-endothelial system. *Nature*, **200**, 503–504
16. Cainzos, M., Potel, J., Puente, J.L. (1988) Anergy in jaundiced patients. *Br.J.Surg.*, **75**, 147–149
17. Greve, J.W., Gouma, D.J., Soeters, P.B. and Buurman, W.A. (1990) Suppression of cellular immunity in obstructive jaundice is caused by endotoxin: a study with germ-free rats. *Gastroenterol.*, **98**, 478–485
18. Roughneen, P.T.M., Drath, D.B., Kulkarni, A.D., Kuma, S.C., Andrassy, R.J. and Rowlands, B.J. (1989) Inflammatory cell function in young rodents with experimental cholestasis: investigation of functional deficits, their aetiology and their reversibility. *J.Pediatr.Surg.*, **24**, 668–673

19. Chatterjee, S.K., Chowdhury, K., Bhattacharya, M. and Barlow, J.J. (1982) Beta-hexosaminidase activities and isoenzymes in normal human ovary and ovarian adenocarcinoma. *Cancer*, **49**, 128–135
20. Steer, C.J., Kusiak, J.W., Brady, R.O. and Jones, E.A. (1979). Selective hepatic uptake of human β-hexosaminidase A by a specific glycoprotein recognition system on sinusoidal cells. *Proc.Natl.Acad.Sci.USA*, **76**, 2774–2778
21. Ullrich, K., Gieselmann, V., Mersmann, G. and Von Figura, K. (1979) Endocytosis of lysosomal enzymes by non-parenchymal rat liver cells: comparative study of lysosomal-enzyme uptake by hepatocytes and non-parenchymal liver cells. *Biochem.J.*, **182**, 329
22. Bearpark, T. and Stirling, J.L. (1977) Clearance of human N-acetyl-β-hexosaminidases from rat circulation. *Biochem.J.*, **168**, 435–439
23. Zuhlsdorf, M., Omort, M., Hasilik, A. and Von Figura, K. (1983) Molecular forms of β-hexosaminidase and cathepsin D in serum and urine of healthy subjects and patients with elevated activity of lysosomal enzymes. *Biochem.J.*, **213**, 733–740
24. Asakawa, H., Hultberg, B., Isaksson, A., Jeppsson, B. and Vagianos, K. (1987) β-hexosaminidase in plasma and liver after partial hepatectomy in normal and cirrhotic rats. *Scand.J.Gastroenterol.*, **22**, 1003–1008
25. Scapa, E., Thomas, P., Loewenstein, M.S. and Zamcheck, N. (1985) Serum β-N-acetyl hexosaminidase (β-NAH) as a discriminant between malignant and benign extrahepatic biliary obstruction: comparison with carcinoembryonic antigen (CEA). *Eu.J.Cancer Clin.Oncol.*, **21**, 1037–1042
26. Hultberg, B., Braconier, J.H., Isaksson, A. and Jansson, B. (1981) β-hexosaminidase level in serum from patients with viral hepatitis as a measure of reticulo endothelial function. *Scan.J.Infect.Dis.*, **13**, 241–245
27. Scapa, E., Thomas, P., Loewenstein, M.S. and Zamcheck, B. (1983) Plasma CEA and β-N-acetyl hexosaminidase (β-NAH) levels as a measure of Kupffer cell function in patients with malignant and benign biliary obstruction. *Gastroenterol.*, **84**, 1394
28. Holstock, G., Leslie, B., Hill, S., Tanner, A.R. and Wright, R. (1982) Monocyte function in cirrhosis. *J.Clin.Pathol.*, **35**, 972–979
29. Pain, J.A. (1987) Reticulo-endothelial function in obstructive jaundice. *Br.J.Surg.*, **74**, 1091–1094
30. Ashendel, C.L. (1985) The phorbol ester receptor: a phospholipid-regulated protein kinase. *Biochem.Biophys.Acta*, **822**, 219–242
31. Scriven, M.W., Thomas, M.A., Manku, M.S., Steward, J.C.M., Horribin, D.F. and Puntis, M.C.A. (1990) Essential fatty acid metabolism is abnormal in obstructive jaundice. *HPB Surg.*, **Suppl 211**
32. Hagve, T.A. (1988) Effects of unsaturated fatty acids on cell membrane functions. *Scand.J.Clin.Lab.Invest.*, **48**, 381–388
33. Puntis, M.C.A., Jiang, W.G. and Hughes, L.E. (1991) Polyunsaturated fatty acids modify in vitro monocyte secretory function in obstructive jaundice. *Br.J.Surg.*, **78**, 1499
34. Hwang, D. (1989) Essential fatty acids and immune response. *FASEB J.*, **3**, 2052–2061
35. Jiang, W.G. and Puntis, M.C.A. (1991) Regulation of monocite lysosomal enzyme secretion by tumour necrosis factor, interleukins and epidermal growth factor. *Gut*, **32**, A1258
36. Puntis, M.C.A., Jiang, W.G., Thomas, M.A., Mathews, N. and Hughes, L.E. (1991) Effect of monocyte production of tumour necrosis factor in jaundice. *Br.J.Surg.*, **78**, 759
37. Virk, K.J., Mahajan, R.C., Dilawari, J.B. and Ganguly, N.K. (1988) Role of β-glucuronidase, a lysosomal enzyme, in the pathogenesis of intestinal amoebiosis: an experimental study. *Trans.Royal Soc.Tropic.Med.Hygiene*, **82**, 422–425
38. Mee, A.S., Nuttall, B.J., Jewell, D.P. (1980) Studies on monocytes in inflammatory bowel disease: factors influencing monocyte lysosomal enzyme activity. *Clin.Exp.Immunol.*, **39**, 785–791
39. Polson, H., Mowat, M.B., Himal, H.S. (1982) Increased mucosal lysosomal enzyme activity in sepsis-associated gastric lesions. *Am.J.Gastroenterol.*, **77**, 457–460

*(Accepted by S. Bengmark 20 January 1993)*

